# Vortioxetine ameliorates anhedonic-like behaviour and promotes strategic cognitive performance in a rodent touchscreen task

**DOI:** 10.1038/s41598-021-88462-7

**Published:** 2021-04-27

**Authors:** Lena-Sophie Martis, Kristoffer Højgaard, Megan C. Holmes, Betina Elfving, Ove Wiborg

**Affiliations:** 1 Department of Clinical Medicine, Aarhus University, Aarhus, Denmark; 2 Centre for Cardiovascular Science, Queen’s Medical Research Institute, University of Edinburgh, Edinburgh, UK; 3 Department of Biomedicine, Aarhus University, Aarhus, Denmark; 4 Centre for Cognitive Ageing and Cognitive Epidemiology, University of Edinburgh, Edinburgh, Scotland UK; 5 Department of Health Science and Technology, Aalborg University, Aalborg, Denmark

**Keywords:** Depression, Phenotypic screening

## Abstract

Depression-associated cognitive impairments are among the most prevalent and persistent symptoms during remission from a depressive episode and a major risk factor for relapse. Consequently, development of antidepressant drugs, which also alleviate cognitive impairments, is vital. One such potential antidepressant is vortioxetine that has been postulated to exhibit both antidepressant and pro-cognitive effects. Hence, we tested vortioxetine for combined antidepressant and pro-cognitive effects in male Long-Evans rats exposed to the chronic mild stress (CMS) paradigm. This well-established CMS paradigm evokes cognitive deficits in addition to anhedonia, a core symptom of depression. Learning and memory performance was assessed in the translational touchscreen version of the paired-associates learning task. To identify the mechanistic underpinning of the neurobehavioural results, transcriptional profiling of genes involved in the stress response, neuronal plasticity and genes of broad relevance in neuropsychiatric pathologies were assessed. Vortioxetine substantially relieved the anhedonic-like state in the CMS rats and promoted acquisition of the cognitive test independent of hedonic phenotype, potentially due to an altered cognitive strategy. Minor alterations in gene expression profiling in prefrontal cortex and hippocampus were found. In summary, our findings suggest that vortioxetine exhibits an antidepressant effect as well as behavioural changes in a translational learning task.

## Introduction

Worldwide, around 264 million people suffer from major depressive disorder (MDD) making this the leading burden of disability worldwide^1^. The recurrent nature of the disease together with insufficient responses to antidepressant treatment add to the devastating burden of the disease^2^. Core symptoms of MDD are a depressed mood and an attenuated anticipation or experience of pleasure (anhedonia). Additionally, patients suffer from a variable number of associated symptoms, such as impaired cognitive abilities, which affect primarily attention, executive functions and memory. These cognitive symptoms persist in 30–60% of treated patients after remission from the affective MDD symptoms. Furthermore, cognitive impairments are the most persisting residual symptoms of depression and, hence, continue to decrease daily functioning and quality of life after remission^3–6^. Moreover, persistent cognitive impairments augment risk of relapse and are increasingly regarded as a core component rather than an epiphenomenon of depression^7,8^. Recovery from cognitive symptoms is associated with a rapid remission from depression^9^, further underlining the importance of restoring cognitive impairments when treating depression.

However, current antidepressant treatment focuses mainly on alleviating the affective symptoms, neglecting cognitive impairments^10^. Therefore, development of novel, pro-cognitive antidepressants is vital and, hence, a translational drug screening platform for depression-associated cognitive impairments is essential. In a previous study^11^, it was demonstrated that the chronic mild stress (CMS) paradigm fulfils exactly these criteria. The CMS model exhibits the MDD core symptom anhedonia (face validity) evoked by stress exposure (etiological validity). Additionally, CMS anhedonic-like rats display depression-associated cognitive impairments, indicated by lower performance in a translational touchscreen learning task, which was not found in CMS resilient, hedonic rats^11^. Hence, cognitive impairments are specific to the depression-like phenotype. In the present study, we follow up by assessing the efficacy of a relatively novel, multimodal antidepressant on affective symptoms and cognitive deficits in the CMS model.

Vortioxetine was approved as an antidepressant in 2013^12^. In addition to an antidepressant action, a pro-cognitive effect was ascribed to vortioxetine due to its multimodal mechanism of action^13^. In MDD patients, executive functions, attention, speed of processing, verbal learning and memory functions, as well as affective symptoms, have been shown to recover after chronic vortioxetine intervention^14^. In rodents, vortioxetine improved spatial working memory, visuo-spatial memory and contextual fear memory besides increasing synaptic plasticity and decreasing behavioural despair^15–19^. Although the CMS model shows high predictive validity for antidepressant actions^20,21^, unexpectedly, vortioxetine was reported to be ineffective in the CMS model^22^. Thus, we investigated, in the present study, if vortioxetine can alleviate the anhedonic-like phenotype of CMS exposed rats using a different route of drug administration. Moreover, cognition of these rats was assessed in the different paired-associates learning (dPAL) touchscreen task, a standardized tool in clinical as well as in preclinical research^23,24^. The rather novel rodent touchscreen platform involves appetitive operant conditioning and was developed based on the human Cambridge Neuropsychological Test Automated Battery (CANTAB); the most frequently applied cognitive assessment tool in depression research^4^. Finally, hippocampal (HPC) and prefrontal cortex (PFC) gene expression was analysed to link neurobehavioral alterations with underlying molecular changes. Genes that are thought to play a role in psychiatric disorders and/or the stress response, such as the mineralocorticoid receptor (*Nr3c2*), glucocorticoid receptor (*Nr3c1*), FK506 binding protein 5 (*Fkbp5*), glycogen synthase kinase 3 beta (*Gsk3b*), disrupted in Schizophrenia 1 (*Disc1*) and brain-derived neurotrophic factor (*Bdnf*) as well as genes important in cognition and neuronal plasticity, such as neuroregulin 1 (*Nrg1*), homer scaffolding protein 1–3 (*Homer1–3*), *Shank 1–3*, *Spinophilin* and *Cofilin 1,* were analysed.

In short, this study aimed to investigate the effect of vortioxetine on the affective state, cognitive performance and cerebral gene expression.

## Materials and methods

### Animals

Male Long-Evans (LE) rats (Janvier Labs, France; *n* = 242) were 5–6 weeks of age weighing 100–120 g at arrival. Rats were single-housed during the experiment with free access to food and water (unless otherwise stated) and kept on a 12-h light–dark cycle. All experiments were conducted according to EU Directive 2010/63/EU, in compliance with the ARRIVE guidelines and approved by the Danish National Committee for Ethics in Animal Experimentation (2013-15-2934-00814).

### Chronic mild stress paradigm

A 1-h sucrose consumption test (SCT, 1.5%) was carried out weekly to assess the hedonic state of each rat throughout the experiment (Supplementary Methods). Following three baseline SCTs, rats were exposed to a number of variable, unpredictable mild stressors in a two-week repeated protocol (Table S1) to provoke a depressive-like phenotype.

After five weeks of CMS, stress exposed rats with a SCT index ≤ 0.7 (average of SCTs in week 4–5 normalised to baseline) were categorized anhedonic-like according to an a priori cutoff^25,26^ and remained in the study.

**Figure 1:**
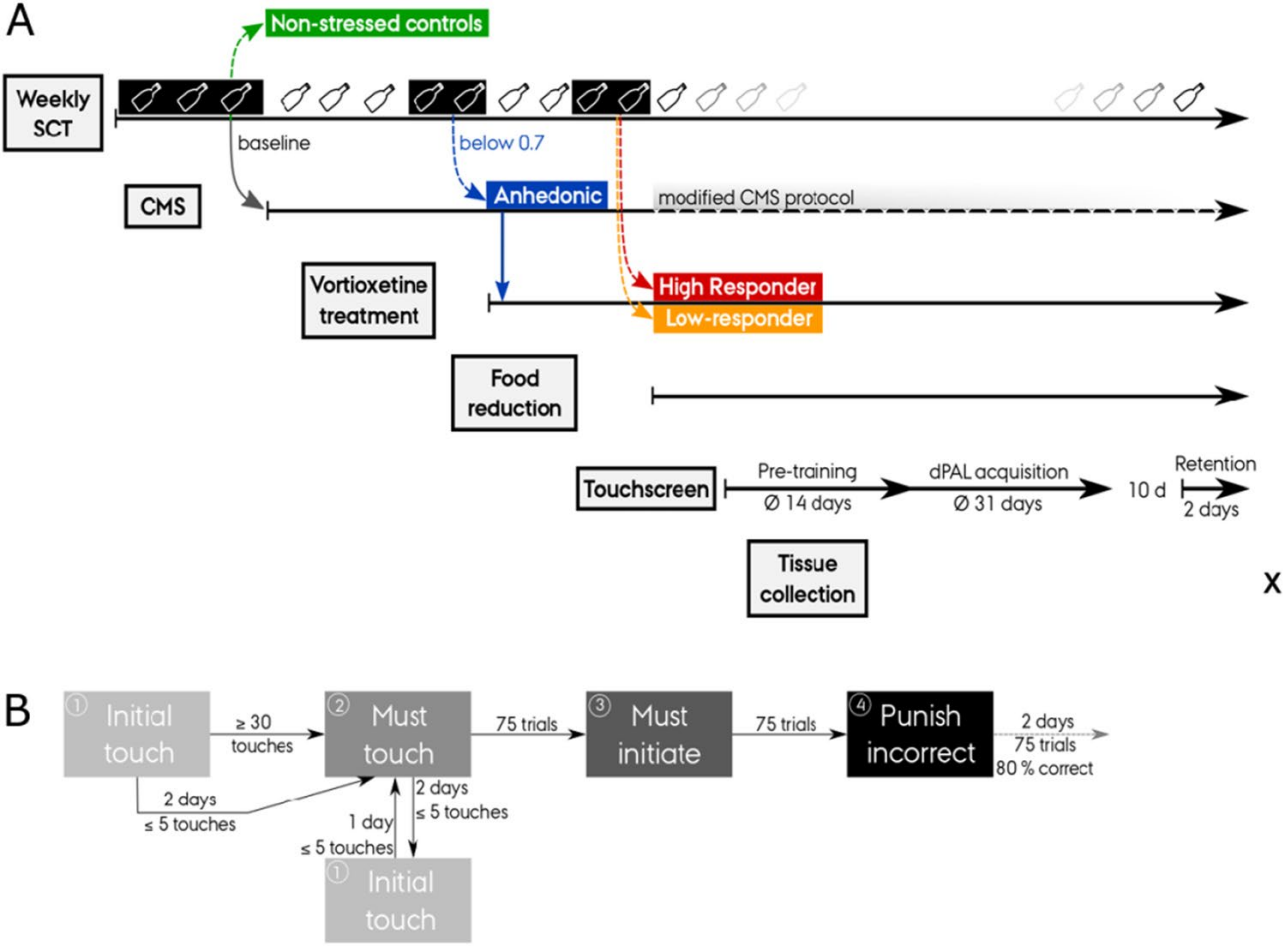
Experimental design. (**A**) Study design. Sucrose consumption tests (SCTs) were conducted throughout the experiment to measure baseline sucrose intake, stress and drug effects (discriminating high vs low respond to treatment). Touchscreen testing included food reduction, pre-training, different paired-associates learning (dPAL) task acquisition and retention. Rats were euthanized and brain tissue was collected (X) 1–3 days after dPAL retention test. (**B**) Touchscreen pre-training. Passing criteria to move on to the next stage are indicated alongside the arrows. Peanut butter was added to the screen when the rat entered “must touch” or when performing ≤ 40 touches in the previous “must touch” session.

Following nine weeks of CMS, which included an initial four weeks of drug treatment, a modified CMS protocol was used (Fig. 1A). Stressors were only applied during the nights reserving daytime for touchscreen assessment. Every Friday, the SCT was carried out followed by 4 h of grouping and light stressors. Thus, touchscreen testing was discontinued on Fridays. The modified CMS schedule (Table S2) was changed every second week to prevent habituation to the milder stress protocol.

### Drug administration

After five weeks of CMS, 45 and 12 anhedonic-like animals were randomly assigned to treatment with vortioxetine or vehicle (Fig. 1A). Group means and standard deviations of the last SCT index before treatment start were comparable for treatment and vehicle group. Standard rat chow (Altromin 1324, Brogaarden, Denmark) was supplemented with vortioxetine (Carbosynth Ltd., UK) at a concentration of 1.8 g/kg rat chow in order to reach a therapeutic dose range with a SERT occupancy above 90%^27^. Following four weeks of treatment combined with CMS, rats were subdivided into high responders (10 rats with highest recovery according to SCT index) and low responders (10 rats with lowest recovery according to SCT index) and subjected to touchscreen testing.

### Touchscreen operant platform

#### Food reduction and touchscreen pre-training

After nine weeks of CMS and four weeks of treatment, 40 rats (control, anhedonic-like, responder, low-responder; *n* = 10/group) were used for touchscreen testing. First, rats were gradually food restricted to 75% of their individual ad libitum consumption (Table S3)^11^. Body weights were monitored daily to ensure rats maintain at least 90% of their body weight during food restriction. Additionally, rats were introduced to peanut butter (Bilka, Denmark) and bacon pellets (45 mg dustless precision pellets, Bio Serv, Flemington, NJ, USA) used for operant conditioning during touchscreen testing. Pre-training was conducted after eight days of food restriction. In four steps, rats were conditioned to operate the touchscreen chamber (Fig. 1B). For further details on pre-training and the Bussey-Saksida touchscreen operant chambers (Campden Instruments Ltd., Loughborough, UK) see Supplementary Method section. Experimenters carrying out behavioural testing were blinded to group identity.

#### Paired-associates learning touchscreen task

Cognitive performance was assessed in the dPAL task, in which a specific symbol-location association needs to be learned. In each trial, only two of the three symbols (spider, flower, plane) would be displayed, one in its correct location (S +) and the other symbol in an incorrect location (S-) on the touchscreen. The third window was left blank (Supplementary Fig. S1). A touch to S + resulted in reward pellet delivery followed by a 20 s inter-trial interval (ITI). Poking S- was followed by a 5 s time out with house light on, the ITI and a correction trial (repetition of the incorrect trial until correct). The six trial types resulting from the stimulus-location association pairs were balanced over the course of a session. dPAL criterion was achieved by completing 75 trials (excluding correction trials) with at least 60 correct trials (≥ 80% accuracy) within 45 min on two consecutive days. Rats that did not acquire the task within 46 session were marked as failing the task by an a priori criterion from a previous study^11^.

#### Retention of the dPAL task

Passing the dPAL task was ensued by a 10-day hiatus without touchscreen testing and an increase in food availability. Rats were then re-tested on the dPAL task for two days to assess long-term memory.

### Cerebral gene expression

A circadian rhythm of BDNF has been reported in certain brain regions^28,29^. Therefore, the rats were sacrificed under similar standardized time conditions from 2 to 4 pm, 1–3 days (*Mean* = 1.3 days) after completing the dPAL retention testing. To diminish a possible effect of the testing, the rats were distributed across the four groups at day 1 to 3. The brain was removed and PFC, dorsal and ventral HPC were dissected and snap frozen on dry-ice. RNA was extracted using the PARIS RNA isolation kit (Ambion, TX, USA). The samples were processed as previously described^30^ and real-time qPCR was performed. A detailed description of RNA extraction and qPCR can be found in Supplementary Methods.

### Statistical analysis

SCT data were analysed by a two-way ANOVA (time x group), followed by group-wise post-hoc comparisons. SCT data are displayed and included in the analysis until the time point when the first animal was terminated after completing the dPAL task.

Summary statistics of the dPAL task (see results section ‘Acquisition of the dPAL task’) were analysed by applying two-way ANOVA (hedonic state x treatment) or by rank aligned two-way ANOVA (indicated with *F*_*rank*_) if assumptions of normality (assessed with QQ-plots) or homogeneity of variance (assessed with Bartlett’s test) were violated. Furthermore, one outlier in the control group for median response latency and two outliers (control and low-responder) for number redundant screen touches were determined by Grubbs (α = 0.05) or ROUT (Q = 1%) test (Prism 7, GraphPad Software Inc., CA, USA) and excluded.

Repeated measurement data analysing learning behaviour across the task (results section ‘Learning phase of the dPAL task’) and learning behaviour within a session (‘Learning behaviour within a single dPAL session’ & Supplementary Results) included all animals (acquiring and failing dPAL acquisition), whereas retention data (‘Long-term memory of dPAL task’ & Supplementary Results) only included animals passing the dPAL task. The data were analysed with repeated measures ANOVA of type III if significant interaction effect was present, otherwise with type II. Mauchly’s sphericity test, if significant, led to Greenhouse–Geisser (GG) (ε < 0.75) or Huynh–Feldt (HF)-corrected repeated measures ANOVA (indicated with *F*_*GG*_ or *F*_*HF*_). Post-hoc comparisons were Bonferroni-corrected. In a separate analysis of memory and relearning performance (Supplementary Results), data were analysed by two-way ANOVA as described in summary statistics.

Normalised target genes were displayed as percent of control group mean (PFC data) or percent of dorsal HPC control mean (dorsal and ventral HPC data) and analysed by two-way ANOVA as described in summary statistics. Differences between dorsal and ventral HPC gene expression were analysed with Student’s *t*-test. Supplementary Table S5 displays *n*-number for each gene and group, thus, the number of outliers removed.

Statistical significance was accepted at *p* < 0.05, two-tailed. Effect size is reported as eta squared (*η*^2^; summary statistics) or generalised eta squared (*η*^2^_*G*_; repeated measures) for cognitive results^31^. All post-hoc comparisons were Bonferroni-corrected. Statistical analyses were performed with RStudio (Version 0.99.892, Boston, USA) and data were displayed with GraphPad Prism 7.

## Results

### Hedonic-like status in response to CMS and vortioxetine treatment

**Figure 2:**
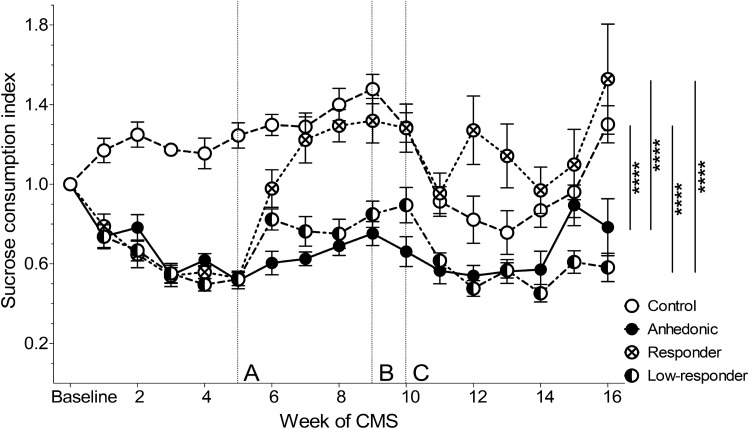
Sucrose consumption test. The consumption index displays the sucrose consumption normalised to baseline sucrose intake prior to CMS exposure. (**A**) Start of antidepressant treatment with vortioxetine. (**B**) Food restriction for touchscreen testing initiated. (**C**) Touchscreen pre-training followed by dPAL acquisition. Group means (± SEM) are displayed. Bonferroni-corrected group comparisons over the entire study are indicated with ****p < 0.0001 (*n* = 10 for all groups).

Following a significant interaction effect of group x time (*F*(45,540) = 5.52, *p* < 0.0001; two-way ANOVA) and main effects of time (*F*(15,540) = 12.82, *p* < 0.0001) and group (*F*(3,36) = 32.24, *p* < 0.0001), Bonferroni-corrected post-hoc analysis revealed that anhedonic-like rats consumed significantly less sucrose during all SCTs compared to non-stressed control rats (*p* < 0.0001). Sixty-five percent of treated rats responded well to vortioxetine and their sucrose intake was not statistically significant different from non-stressed controls, but significantly increased compared to untreated, anhedonic-like rats (*p* < 0.0001). Rats that responded poorly to vortioxetine, thus low-responders, consumed significantly less sucrose than responders (*p* < 0.0001) or non-stressed controls (*p* < 0.0001), but were not statistically significantly different to anhedonic-like rats (Fig. 2).

### Paired-associates learning touchscreen task

#### Acquisition of the dPAL task

**Figure 3:**
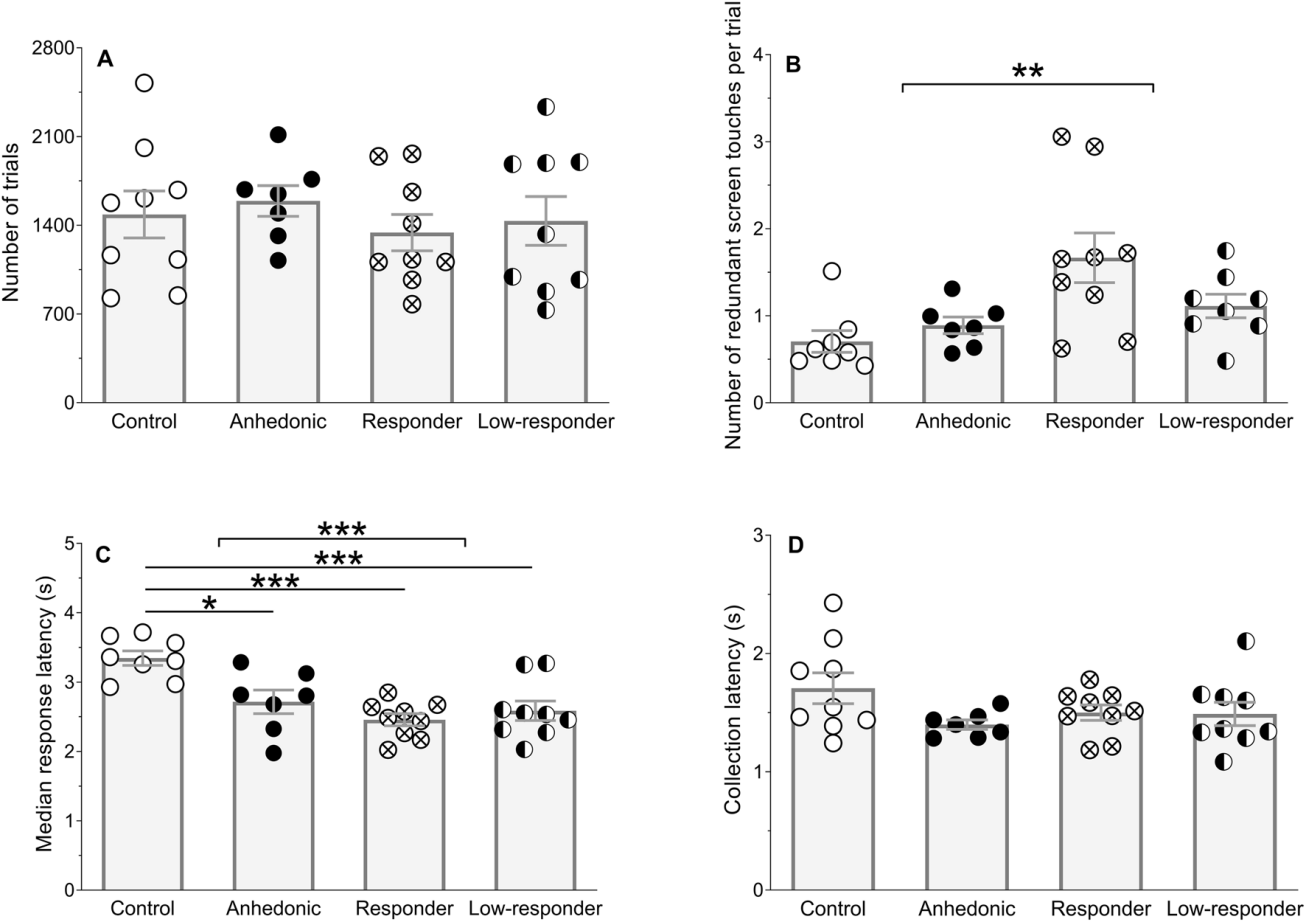
Acquisition of dPAL task. (**A**) The accumulated number of trials needed to acquire the dPAL task. (**B**) The number of additional, i.e. redundant screen touches per trial (trial or correction trial) averaged across all sessions for each animal. (**C**) Median response latency to touchscreen stimuli averaged across all sessions. (**D**) Reward collection latency averaged across all sessions. Only animals, which acquire the dPAL task, are analysed and displayed as individual data points and group means (± SEM). Two-way ANOVA main effects and Bonferroni post-hoc comparisons are indicated by *p < 0.05, **p < 0.01, ***p < 0.001.

Acquisition of the dPAL task, indicated by the accumulated number of trials over all sessions to reach criterion for passing, did not differ significantly between groups (Fig. 3A).

Two-way ANOVA revealed that drug treatment increased the number of redundant screen touches compared to untreated animals (main effect of treatment: *F*(1,28) = 9.98, *p* = 0.004, *η*^2^ = 0.23). This treatment effect is possibly driven by a trend in hedonic state x treatment interaction effect (*F*(1,28) = 1.12, *p* = 0.063, *η*^2^ = 0.08), i.e. responders diverging (Fig. 3B).

Median response latency was altered due to a hedonic state x treatment interaction effect (*F*(1,29) = 9.03, *p* = 0.005, *η*^2^ = 0.15; Fig. 3C). Specifically, anhedonic-like rats (*p* = 0.013), responders (*p* = 0.0001) and low-responders (*p* = 0.001) responded faster to touchscreen stimuli than non-stressed control rats. Furthermore, treatment alone reduced median response latency (*F*(1,29) = 17.58, *p* = 0.0002, *η*^2^ = 0.30; Fig. 3C).

There was no difference in reward collection latency (Fig. 3D) or number of correction trials between groups.

Six animals (one non-stressed control, three anhedonic-like rats, one responder and one low-responder) did not pass dPAL and, thus, were excluded from this analysis.

#### Learning phase of the dPAL task

To compare learning curves with repeated measures ANOVA, the rats’ variable number of sessions and trials per session was normalised^32^. Thus, for each rat, the total number of trials (trials + correction trials) to learn the dPAL task was split into ten equal bins^11^.

**Figure 4:**
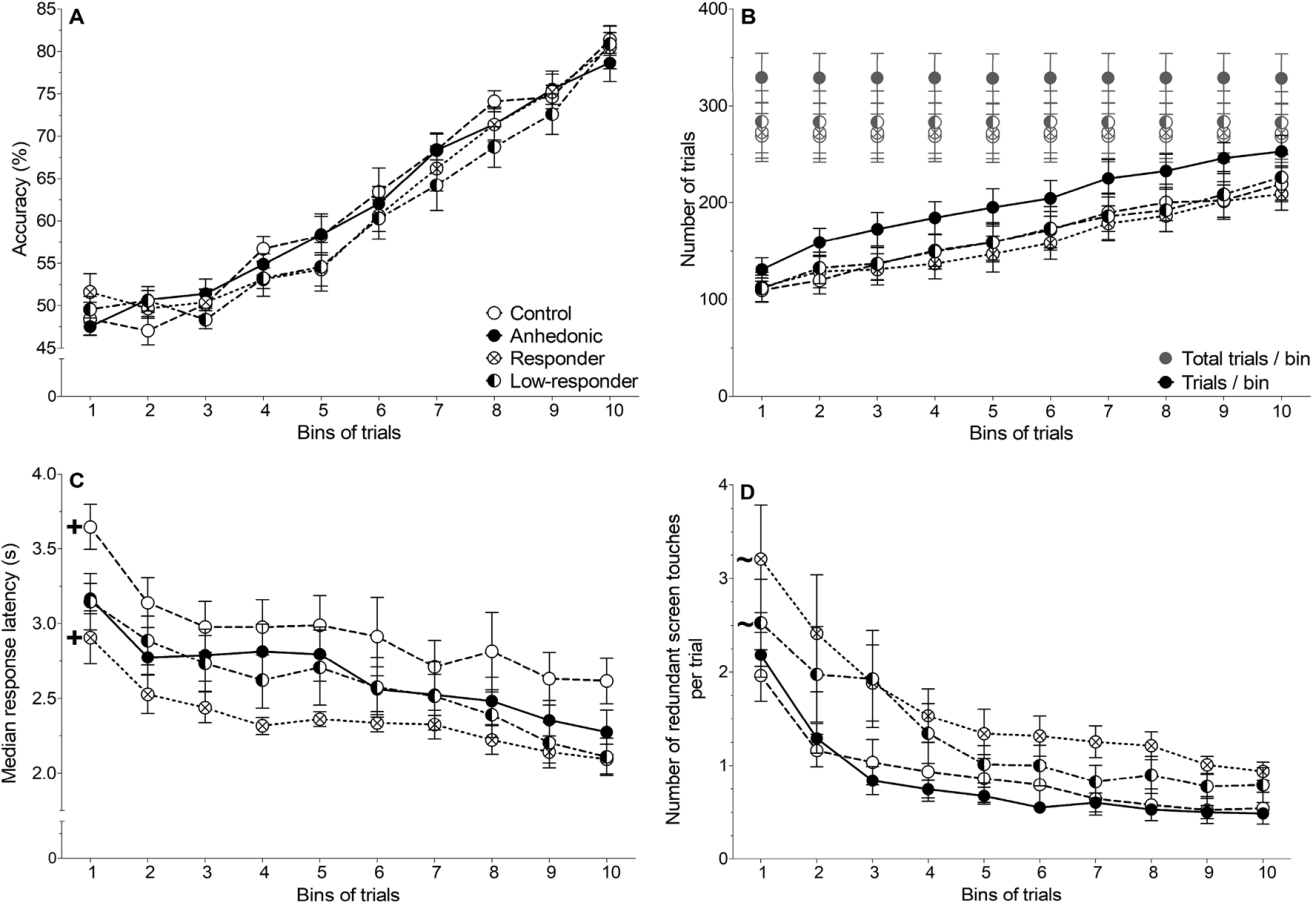
Behavioural parameters during dPAL task acquisition of all animals. (**A**) Accuracy; percent of correct choices. (**B**) Number of trials (black) and number of total trials (trials plus correction trials, grey). (**C**) Median response latency. (**D**) Number of additional, i.e. redundant screen touches per trial (trial or correction trial). Group means (± SEM) are shown with ‘ + ’ indicating a significant difference of the respective group to the three other groups and ‘ ~ ’ indicating a significant difference to controls and anhedonic-like rats (Bonferroni post-hoc comparisons; *n* = 10 for all groups).

The percentage of correct trials (accuracy) increased significantly over time, thus, with increasing number of bins (*F*_*GG*_(3.00,107.98) = 30.08, *p* < 0.0001, *η*^2^_*G*_ = 0.08), indicating task learning. No effect of group on accuracy was observed (Fig. 4A).

The number of trials performed increased significantly over time with growing bin number (*F*_*GG*_(3.08,110.85) = 47.90, *p* < 0.0001, *η*^2^_*G*_ = 0.10), whereas the number of correction trials decreased significantly by bin number (*F*_*GG*_(3.08,110.73) = 48.37, *p* < 0.0001, *η*^2^_*G*_ = 0.17; Fig. 4B). This also indicates learning of the task, however, no statistically significant differences between groups were observed.

Control rats responded slower to stimuli compared to anhedonic-like rats (*p* = 0.002), low-responders (*p* < 0.0001) or responders (*p* < 0.0001). However, vortioxetine responders showed the shortest median response latency compared to controls, anhedonic-like rats (*p* = 0.002) and low-responders (*p* = 0.029; main effect of group: *F*(3,36) = 3.24, *p* = 0.033, *η*^2^_*G*_ = 0.15). The median response latency decreased significantly during dPAL acquisition (main effect of time: *F*_*GG*_(4.32,155.40) = 9.14, *p* < 0.0001, *η*^2^_*G*_ = 0.08; Fig. 4C).

Vortioxetine responders executed the highest number of redundant screen touches per trial compared to control rats (*p* < 0.0001) and anhedonic-like rats (*p* < 0.0001). Vortioxetine low-responders also performed more redundant screen touches than control (*p* = 0.022) and anhedonic-like rats (*p* = 0.005; main effect of group: *F*(3,36) = 3.10, *p* = 0.039, *η*^2^_*G*_ = 0.13). The number of redundant screen touches decreased during dPAL acquisition (main effect of time: *F*_*GG*_(2.46,88.45) = 5.67, *p* < 0.0001, *η*^2^_*G*_ = 0.06; Fig. 4D).

Collection latency was not significantly different between groups or over time, suggesting equal motivation for reward collection and for engaging in the dPAL task.

#### Learning behaviour within a single dPAL session

Every single session of an animal was divided into six blocks by reference to the total number of trials (trials + correction trials). Average performance per block was determined for each animal. This allowed analysis of learning behaviour within the time course of a session.

During a session, accuracy did not change significantly over time, nor between groups. The number of trials executed during a session changed depending on session block (main effect of session block: *F*(5,180) = 3.38, *p* = 0.006, *η*^2^_*G*_ = 0.02; Supplementary Fig. S2A). For further details see Supplementary Result Section.

#### Long-term memory of dPAL task

Long-term memory performance was assessed by re-testing rats in dPAL following a 10-day hiatus after dPAL acquisition. Included in the analysis was accuracy of the last session of dPAL acquisition before the break as well as the two dPAL retention sessions after the break. A trend of an interaction effect of group x session (*F*_*GG*_(4.28,42.75) = 2.10, *p* = 0.066, *η*^2^_*G*_ = 0.05) and a main effect of time (*F*_*GG*_(1.43,42.75) = 8.91, *p* = 0.0004, *η*^2^_*G*_ = 0.36) on accuracy was observed. Bonferroni post-hoc comparisons revealed that all groups decreased accuracy of task retention in session one, vortioxetine responders significantly increased their accuracy on retention session two and all groups continued to show a lower accuracy on session two compared to passing criterion (Supplementary Fig. S3A). For further details see Supplementary Result Section.

### Cerebral gene expression

**Figure 5:**
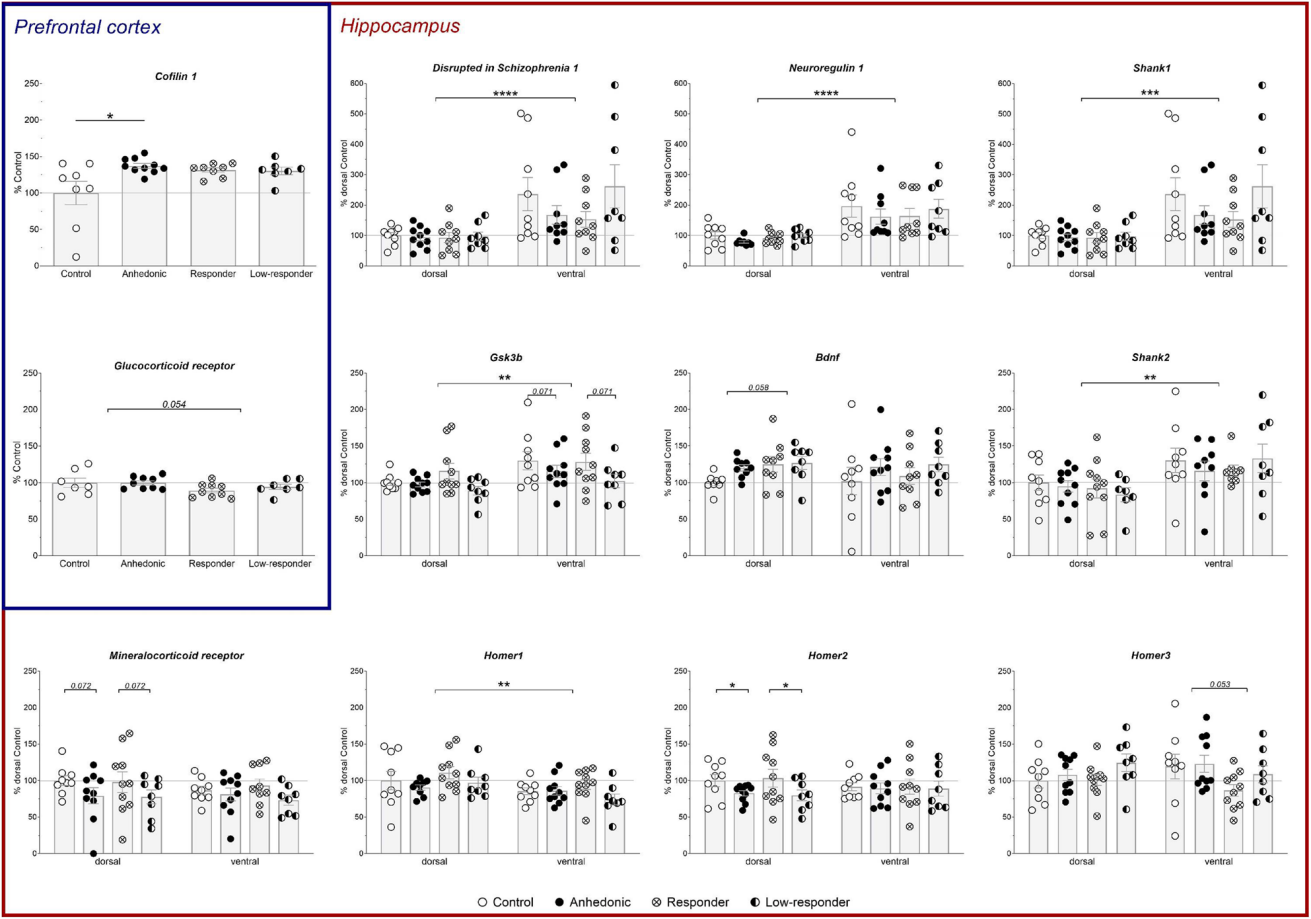
Prefrontal cortex (PFC) and hippocampal (HPC) gene expression levels. Genes of interest are normalised to reference genes and displayed as percent of control mean for the PFC or as percent of the control mean of the dorsal HPC for ventral and dorsal HPC tissue. Individual data points as well as group means (± SEM) are displayed. Statistical significance is indicated for main effects and between tissue differences (angular brackets), and Bonferroni corrected post-hoc comparisons by ****p < 0.0001, ***p < 0.001, **p < 0.01, *p < 0.05; and trends by the respective number. *Bdnf*—Brain-derived neurotrophic factor; *Disc1*—Disrupted in Schizophrenia 1; *Gsk3b*—Glycogen synthase kinase 3 beta; *Homer*—Homer scaffolding protein; *Nr3c1*—Glucocorticoid receptor; *Nr3c2*—Mineralocorticoid receptor; *Nrg1*—Neuroregulin 1.

Alterations in gene expression levels were analysed in response to vortioxetine treatment and hedonic state. Furthermore, differences between dorsal and ventral HPC gene expression were examined. Regulated genes are presented in Fig. 5. Supplementary Table S5 contains all gene expression levels for the four groups and all tissues.

#### Prefrontal cortex gene expression

In the PFC, the expression level of *Cofilin 1* was increased in the anhedonic-like group (*p* = 0.022) compared to controls (interaction effect of hedonic state x treatment: *F*_*rank*_(1,28) = 5.51, *p* = 0.026). A trend of treatment reducing expression of *Nr3c1* mRNA was observed (*F*(1,27) = 4.07, *p* = 0.054). The mRNA expression levels of *Nr3c2, Fkbp5*, *Disc1*, *Gsk3b*, *Bdnf*, *Shank 1–3*, *Homer1–3*, *Nrg1,* and *Spinophilin* were not affected.

#### Hippocampal gene expression

The gene expression of *Gsk3b, Disc1, Shank1, Shank2*, and *Nrg1* was higher in the ventral compared to dorsal HPC (*t*(35) =  − 3.13, *p* = 0.004; *t*(34) =  − 4.72, *p* < 0.0001; *t*(34) =  − 3.99, *p* = 0.0003; *t*(32) = − 3.58, *p* = 0.001; and *t*(32) = − 5.84, *p* < 0.0001, respectively). For *Homer1* the expression was decreased in the ventral compared to the dorsal HPC (*t*(35) = 3.01, *p* = 0.005).

In the dorsal HPC, *Homer2* gene expression was decreased in groups with anhedonic-like phenotype (main effect of hedonic state: *F*(1,33) = 5.63, *p* = 0.024; Fig. 5).

Close to significant trends due to treatment and/or hedonic state were observed for *Nr3c2, Disc1, Gsk3b, Bdnf* and *Homer3* mRNA levels (Fig. 5; statistics in Table S5); with no notable observations on *Fkbp5*, *Nr3c1*, *Shank3, Spinophilin*, or *Cofilin* *1* gene expression across tissues, hedonic state or treatment.

## Discussion

In the present CMS study, non-stressed controls, anhedonic-like rats and vortioxetine treated rats were assessed for hedonic state, cognitive performance and cerebral gene expression profiling.

### Vortioxetine recovers the hedonic state

CMS exposed rats decreased sucrose intake over time, indicating a reduced reward sensitivity and, hence, mirroring the MDD core symptom anhedonia. Administration of the antidepressant vortioxetine recovered the hedonic state in a major fraction of anhedonic-like rats (65%), whereas the remaining rats responded poorly and remained in an anhedonic-like state. Previously vortioxetine was reported to be ineffective when tested in the CMS model^22^. However, vortioxetine was administered by intraperitoneal injections once daily (Mariusz Papp, personal communication), and the relatively short half-life of vortioxetine in rodents^22^ may explain for the ineffective treatment outcomes in this study. In the present study vortioxetine was mixed into the diet and, hence, this route of drug administration ensured a more even and continuous diurnal drug exposure. In a parallel study using the same dose and route of administration, we confirmed comparable vortioxetine serum levels (unpublished data) as shown to be therapeutically relevant^27,33^. Food restriction necessary for touchscreen training likely resulted in a slightly reduced dose of vortioxetine. However, this reduction was comparable across animals and groups (Suplementary Fig. S5). Furthermore, monitoring of the hedonic state with SCTs throughout the study (Fig. 2) showed that vortioxetine responders remained comparable to controls and above the criterion for anhedonia even during food reduction.

### Vortioxetine affects cognition

In the present study we also investigated whether vortioxetine-induced alleviation of the hedonic state is associated with alterations in cognitive performance. Vortioxetine has been reported to augment cognitive functions^22^ and is believed to be a directly mediated effect rather than caused through remission from affective symptoms^14^. In the present study, vortioxetine did not alter primary touchscreen parameters (accuracy, number of trials) compared to non-stressed controls or anhedonic-like rats. However, we noticed that three out of ten anhedonic-like rats did not pass the dPAL task within 46 sessions whereas only one animal failed to pass in any of the other groups. This observation might be attributed to normal biological variation considering the small group size (*n* = 10). Alternatively, the inability to acquire the dPAL task might suggest cognitive impairment in the anhedonic-like group and, consequently, a potential pro-cognitive effect of vortioxetine treatment. Future studies are needed to validate this interpretation.

Importantly, the latency for collecting reward pellets did not differ between groups. This suggests equal incentive to consume the reward and presumably to participate in the touchscreen task. Likely, this behaviour is driven by hunger due to the food restriction accompanying touchscreen testing^11^.

Consistently, median response latency was reduced in all CMS-exposed groups compared to controls. During task acquisition, vortioxetine responders displayed the shortest median response latency and controls the longest latency. Prolonged median response latency in the control group is consistent with a previous study^25^, suggesting increased cognitive appraisal, before executing a choice in control animals. Consequently, reduced response latency in the anhedonic and mainly in the vortioxetine treated groups can be considered as impulsive behaviour, executing a less evaluated, spontaneous choice. Reduced response latency may indicate impaired HPC functioning since inactivation of the dorsal HPC with lidocaine and scopolamine significantly shortened reaction time in the rat dPAL task as well^34^ and is in line with the important role of HPC in visuospatial learning tasks^32,35^. An alternative explanation might include a frontostriatal reorganization causing a shift from effortful, goal-directed to habitual behaviour. Such changes have been observed after stress exposure^36^ and might explain the reduced response latency observed in the present study. Noticeably, responders to vortioxetine treatment displayed the shortest response latency of all groups suggesting an association between treatment response and decreased appraisal.

A shift to habit-like or impulsive behaviour is further supported by the number of redundant screen touches per trial. Consistently, vortioxetine treated rats executed more redundant touches than any other group. Thus, vortioxetine seems to increase impulsive or compulsive behaviour. This lack of inhibitory control may suggest impairments in executive function associated with the PFC^37^.

In order to address long-term memory, accuracy was re-tested after a 10-day hiatus subsequent to passing dPAL. Vortioxetine responders decreased most in accuracy after the 10-day hiatus and performed significantly worse than low-responders. Hence, a high response to vortioxetine treatment was associated with reduced memory performance. Interestingly, only the control group restored performance to the dPAL passing criterion level (≥ 80% accuracy) on the second day of retention. All other groups still performed below 80% accuracy and the anhedonic-like group even decreased in accuracy on the second day of retention.

### Altered cerebral gene expression associated with vortioxetine treatment and hedonic state

Expression levels of genes regulated in neuropsychiatric diseases or associated with neuronal plasticity were measured in the PFC, dorsal and ventral HPC. Cofilin 1 is a key regulator in growth cone dynamics and, thus, in neuronal plasticity important for learning and memory^38,39^. In the PFC*, Cofilin 1* expression was upregulated in anhedonic-like rats compared to controls. Excessive up- or down-regulation of *Cofilin 1* was associated with impaired synaptic plasticity and learning deficits^39^. Thus, altered *Cofilin 1* gene expression might suggest subthreshold cognitive impairments associated with anhedonia, especially in untreated rats.

DISC1 is a scaffolding protein involved in neurodevelopmental signalling and suggested as candidate gene in neuropsychiatric disorder^40,41^. In the present study, *Disc1* gene expression levels were higher in the ventral compared to the dorsal HPC. In the ventral HPC, an interaction trend may indicate a regulatory association of the hedonic state and vortioxetine treatment on *Disc1* gene expression. These changes support the literature that DISC1 dysregulation is involved in the pathology of mental illnesses including cognitive deficits and dendritic arborisation^42,43^.

DISC1 regulates downstream *Gsk3b* expression and in the present study, *Gsk3b* expression was upregulated in the ventral compared to the dorsal HPC, which might be linked to an increased *Disc1* gene expression. *Gsk3b* expression is known to be inhibited by most antidepressant treatments, e.g. SSRIs, and a dysregulation of *Gsk3b* expression is suggested to be implicated in depression^44–47^. *Gsk3b* upregulation is associated with impairments in spatial memory, attention and long-term potentiation, which are all important elements in acquisition of the dPAL task^48–52^. Consequently, borderline increased *Gsk3b* gene expression levels in the dorsal HPC in the present study may underlie the observed memory impairments during dPAL retention in the vortioxetine responder group compared to low-responders.

Homer proteins, which are scaffolding proteins facilitating post-synaptic signalling, are vital for learning and memory functions^53^. Moreover, decreased *Homer1* expression is associated with an enhanced stress response and susceptibility to psychiatric diseases such as MDD^54,55^. In the present study, *Homer1* was higher expressed in the dorsal than in the ventral HPC, possibly in response to spatial learning required for dPAL acquisition^56^. In the dorsal HPC, *Homer2* mRNA expression was decreased in rats with anhedonic phenotype (treated and untreated). *Homer2* is required for alcohol-seeking^57^ and, thus, reduced seeking of reward in anhedonic-like rats may be reflected by decreased *Homer2* levels. Although *Homer3* was upregulated in rat frontal cortex in response to vortioxetine treatment (not correcting of multiple comparisons)^58^, only a trend of vortioxetine downregulating *Homer3* expression in the ventral hippocampus was observed in the present study.

*Bdnf* is involved in neuronal plasticity^59^, a mechanism which might be upregulated by vortioxetine treatment^58^. Moreover, *Bdnf* expression levels are reduced following stress exposure as well as in PFC and HPC *post-mortem* tissue of MDD suicide victims^60,61^. Furthermore, antidepressant treatment elevates *Bdnf* levels and, in turn, treatment efficacy appears dependent on *Bdnf* levels^62–64^. Consequently, the trend of higher *Bdnf* levels in the dorsal HPC of vortioxetine treated animals is in accordance with the literature.

NR3C2 expression is an important player in the stress response, HPA axis activity and MDD. Increased NR3C2 function is associated with resilience, whereas decreased NR3C2 levels suggest stress-susceptibility for developing depression^65^. Hence, the anhedonic phenotype, i.e. susceptibility to CMS including a low treatment response to vortioxetine, might be linked to a reduced *Nr3c2* expression in the HPC.

In future studies, it would be interesting to include gene expression profiling before start of behavioural testing as well as after or, alternatively, a behaviourally naïve, vortioxetine-treated group can be added to disentangle the effects of the learning paradigm from treatment effects.

### Touchscreen testing

To our knowledge, this was the first touchscreen study to show that not only sweet rewards, such as sugar pellets or milkshakes, generate successful operant conditioning. This might become crucial in addiction, diabetes or reward studies and expands the applicability of touchscreen testing. Furthermore, continuous SCTs throughout the experiment revealed the impact of food reduction, treatment and appetitive touchscreen testing on rodents.

## Conclusion

Our study expands on the relatively new drug treatment approach of antidepressants targeting depression-associated cognitive impairments. Hence, the effect of vortioxetine on the hedonic state, on cognition and selected gene expression was assessed. In contrast to a previous report (reviewed in Sanchez et al.^22^), we have shown that vortioxetine recovers the hedonic state in anhedonic-like rats and, hence, demonstrated its efficacy in a well-validated preclinical model of depression^26,66,67^. Moreover, cognitive performance was assessed with the touchscreen operant platform, which was developed with focus on its translational value. In the present study, the primary readouts did not reveal beneficial cognitive effects of vortioxetine treatment although it was observed that a higher number of treated rats managed to pass the dPAL task. Furthermore, effects on behavioural strategy was evident from secondary read-outs. The potential pro-cognitive effect of vortioxetine requires more detailed evaluation since the observed effects, such as shortened reaction time and a shift to habitual behaviour might be beneficial in a different context than what the dPAL touchscreen task is actually designed for addressing. Finally, the most pronounced alterations in the selected genes were in the dorsal versus the ventral HPC. However, it cannot be excluded that the learning paradigm has affected the gene expression profiles.

## Supplementary Information

Supplementary information
