# Supplementary material for: Vortioxetine ameliorates anhedonic-like behaviour and promotes strategic cognitive performance in a rodent touchscreen task
Source: Sci Rep. 2021 Apr 27;11:9113. doi: 10.1038/s41598-021-88462-7 (PMC8079376; doi:10.1038/s41598-021-88462-7)
Supplement: Supplementary file 1 — Supplementary information [file 41598_2021_88462_MOESM1_ESM.pdf]

Vortioxetine ameliorates anhedonic-like behaviour and promotes strategic cognitive performance in a rodent touchscreen task

Lena-Sophie Martis[a,b], Kristoffer Højgaard[a,c], Megan C. Holmes[b,d], Betina Elfving[a], Ove Wiborg[e]

a Department of Clinical Medicine, Aarhus University, Denmark

b Centre for Cardiovascular Science, Queen's Medical Research Institute, University of Edinburgh, United Kingdom

c Department of Biomedicine, Aarhus University, Denmark

d Centre for Cognitive Ageing and Cognitive Epidemiology, University of Edinburgh, Scotland, United Kingdom

e Department of Health Science and Technology, Aalborg University, Denmark

Supplementary Section

Supplementary Methods and Materials

Sucrose consumption test

A 1-h sucrose consumption test (SCT, 1.5%) was carried out weekly to assess the hedonic state of each rat. After three baseline measurements, rats were split into a control and CMS group (same baseline group mean and *SD*). Stress exposure for the latter group commenced immediately after the last SCT baseline acquisition. Weekly SCTs were continued throughout the experiment. SCT indexes (SCT normalised to baseline) were used to determine CMS susceptibility, i.e. anhedonic-like rats, and high vs low responders to antidepressant treatment (Figure 1A).

Touchscreen Apparatus

The sound- and light-attenuating Bussey-Saksida touchscreen operant chambers (Campden Instruments Ltd., Loughborough, UK) contained a trapezoid shaped interior (height 300 mm, length 332 mm, width screen 240 mm, width magazine 126 mm). A touch-sensitive screen was located at the wide side of the interior box and a reward delivery system (magazine) at the opposite wall. A mask covered the

touchscreen leaving only three windows (100 x 60 mm) for the rat to touch the screen. A fan ensured sufficient ventilation and masking of external noise. The chambers were further equipped with a grid floor, house and magazine light, and a tone generator. The touchscreen program was controlled by Whisker Server and Abett II software (Campden Instruments Ltd.).

Touchscreen pre-training

Each rat was tested once daily Saturday - Thursday with a session lasting max 45 min or 75 trials. To prevent touchscreen chamber-specific effects, groups were balanced across chambers. Daytime-specific biases were prevented by testing rats from all groups in parallel and rotating testing order[1]. Rats were habituated to the testing room for 30 min. In the first pre-training step, "initial touch", rats received automatically a bacon pellet after 30 s of stimulus presentation (randomly one of the three windows was illuminated). Three instead of one reward pellet would appear if the rat touched the screen during stimulus presentation. Reward pellet dispensation always coincided with stimuli disappearance, a tone and food magazine illumination throughout touchscreen assessment. After reward collection a 20 s inter-trial-interval (ITI) started. A new trial followed. Rats passed "initial touch" by executing 30 or more screen touches. Rats, which failed to touch the screen, were also moved on to the next step to encourage them in an active participation and with the option to return them to "initial touch" (Figure 1B). Daily food was increased to 80% of *ad libitum* intake for consequent touchscreen stages. In "must touch", rats had to touch the screen to receive one reward pellet. Rats passed by completing 75 screen touches, thus 75 trials, within 45 min. Rats with a low number of trials were moved back to "initial touch" (if they had not passed "initial touch" beforehand) to increase their motivation (Figure 1B). In the third step "must initiate", rats had to initiate each trial after the ITI by poking into the illuminated food magazine. Otherwise, the trial followed the concept of "must touch". Finally, in "punish incorrect", rats only received the reward touching the illuminated window. A touch to a blank window was punished by a 5 s time out interval with house light on and followed by the ITI. Such an incorrect trial was ensued by a "correction trial" in which the same window as before was illuminated. Passing "punish incorrect" by completing 75 trials (excluding correction trials) within 45 min with at least 60 correct choices ($\geq$ 80% accuracy) on two consecutive days equalled the end of pre-training.

RNA extraction and cDNA synthesis

Tissue was stored at -80 °C until extraction of RNA with the PARIS RNA isolation kit (Ambion, TX, USA). The samples were processed as previously described[2]. The RNA concentration and the purity were determined by a NanoDrop 1000 spectrophotometer (ThermoFisher Scientific, Delaware, USA). RNA was reversely transcribed using random primers and Superscript IV Reverse Transcriptase (Invitrogen,

CA, USA) following manufacturer's instructions and with a RNA input concentration per reaction of 129 ng/µl and 12.8 ng/µl for PFC and HPC, respectively.

Real-time qPCR

Real-time qPCR was carried out on individual samples in 96-well PCR-plates using the Mx3005P (Stratagene, La Jolla, CA, USA) and SYBR Green as described previously[3]. The gene expression of eight different reference genes (*18s rRNA, ActB, CycA, Gapd, Hmbs, Hprt1, Rpl13A*, and *Ywhaz*) and 15 different target genes (*Nr3c1, Nr3c2, Bdnf, Fkbp5, Disc1, Gsk3b, Nrg1, Shank1-3, Homer1-3*, *Spinophilin,* and *Cofilin 1*) were investigated. Primer sequences and amplicon sizes are given in Table S4. The thermal conditions for the PCR were 3 min at 95 °C, followed by 40 cycles of 10 s denaturation at 95 °C, 30 s annealing at 60 °C, and 60 s extension at 72 °C. Stability comparison of the expression of the eight reference genes was conducted with the Normfinder software[4] and the best combination selected. Values for each individual were normalized with the geometric mean of the reference genes *ActB* and *Rpl13A* (PFC) as well as *Hprt* and *CycA* (HPC), respectively.

Supplementary Results

Learning behaviour within a single dPAL session

Non-stressed controls needed significantly less correction trials within a session compared to responders ($p < 0.0001$) and low-responders ($p < 0.0001$) and a trend to anhedonic-like rats ($p = 0.054$). Anhedonic-like rats needed significantly less correction trials than vortioxetine responders ($p = 0.015$) and showed a trend to low-responders ($p = 0.063$; main effect of group: $F(3,36) = 3.05$, $p = 0.041$, $\eta^2_G=0.16$; Supplementary Fig. S2A). The number of correction trials significantly decreased by session block (main effect of session block: $F(5,180) = 3.71$, $p = 0.003$, $\eta^2_G=0.02$) indicating learning during an individual session.

Vortioxetine responders executed more redundant touches per trial (correction or non-correction trial) than the control group ($p < 0.0001$) or anhedonic-like rats ($p < 0.0001$), but not significantly more than low-responders. Low-responders carried out more redundant screen touches than control ($p = 0.020$) or anhedonic-like rats ($p = 0.005$; main effect of group: $F(3,36) = 3.12$, p = 0.038, $\eta^2_G=0.19$; Supplementary Fig. S2B). The number of redundant touches decreased significantly during a session (main effect of session block: $F_{GG}(1.73,62.22) = 9.65$, $p < 0.001$, $\eta^2_G=0.03$).

Non-stressed controls took longer to execute their choice, as observed by median response latency, than anhedonic-like rats ($p = 0.010$), responders ($p < 0.0001$) or low-responders ($p < 0.0001$;

main effect of group: $F(3,36) = 4.15$, $p = 0.013$, η^2_G=0.15; Supplementary Fig. S2C). Median response latency increased within a session (main effect of session block: $F_{GG}(1.68,60.44) = 9.57$, $p < 0.0001$, η^2_G=0.12).

Collection latency was not significantly different between groups and independent of session block.

Long-term memory performance

Additionally, individual changes in accuracy due to memory (difference in accuracy between last dPAL acquisition session and first retention session) and relearning (difference in accuracy between the two retention sessions) were evaluated. The hedonic state significantly associated with memory performance ($F(1,30) = 5.19$, $p = 0.030$, η^2=0.13; Supplementary Fig. S3B) and a trend in hedonic state x treatment on memory was observed ($F(1,30) = 3.06$, $p = 0.090$, η^2=0.08). Although statistically valid, the association of hedonic state and memory was unexpected examining the similar mean group performances (Supplementary Fig. S3B). Thus, a one-way ANOVA was performed to extract group difference in memory performance. Memory performance was significantly different between groups ($F(3,30) = 3.41$, p=0.030, η^2=0.25). Treatment responders (-13.56 ± 5.65%) showed a lower memory performance compared to low-responders (-6.74 ± 6.04%, Bonferroni p=0.046). No effect of group was found for relearning performance following neither two-way nor one-way ANOVA (Supplementary Fig. S3B).

References

1. Martis, L.-S. *et al.* The effect of rat strain and stress exposure on performance in touchscreen tasks. *Physiol. Behav.* **184**, 83–90 (2018).

2. Müller, H. K., Wegener, G., Popoli, M. & Elfving, B. Differential expression of synaptic proteins after chronic restraint stress in rat prefrontal cortex and hippocampus. *Brain Res.* **1385**, 26–37 (2011).

3. Elfving, B., Christensen, T., Ratner, C., Wienecke, J. & Klein, A. B. Transient activation of mTOR following forced treadmill exercise in rats. *Synapse* **67**, 620–625 (2013).

4. Andersen, C. L., Jensen, J. L. & Ørntoft, T. F. Normalization of Real-Time Quantitative Reverse Transcription-PCR Data: A Model-Based Variance Estimation Approach to Identify Genes Suited for Normalization, Applied to Bladder and Colon Cancer Data Sets. *Cancer Res* **64**, 5245–5250 (2004).

Table S1. Two-week chronic mild stress (CMS) protocol. Stressors and duration. During "grouping", one CMS rat (intruder) was introduced to the home cage of another CMS rat (resident). Acting as resident or intruder as well as grouping partners were alternated weekly.

	Monday	Tuesday	Wednesday	Thursday	Friday	Saturday	Sunday
**Morning**	New cage*, intermittent illumination (5 h)	Water deprivation (9 h)	Stroboscopic light (6 h)	New cages	SCT (1 h)*, food or water deprivation⁺ (7 h)	Water or food deprivation⁺ (7 h)	Cage tilted 45° (9 h)
**Evening**		Cage tilted 45° (14 h)	Wet bedding (14 h)	Remove food and water (14 h)*	Grouping (14 h)	Cage tilted 45° (14 h)	Wet bedding (14 h)

*For CMS and non-stressed controls, ⁺Alternating weekly.

Table S2. Modified chronic mild stress (CMS) protocol during touchscreen pre-training and acquisition. Stressors were applied during the night phase since touchscreen testing took place during the day. On Fridays, the SCT (including all animals) followed by grouping (only CMS animals) were carried out during the light phase.

Protocol	Monday	Tuesday	Wednesday	Thursday	Friday	Saturday	Sunday
A	Strobe light (6 h)	Wet bedding (14 h)	Light on (3 x 2 h)	Remove food and water (15 h)*	SCT (1 h)*, Grouping (4 h), Strobe light (2 x 2 h) and light on (1 h)	Cage tilted 45° (14 h)	Wet bedding (14 h)
B	Cage tilted 45° (14 h)	Light on (2 h), strobe light (5 h)	Light on (3 x 2 h, 1h)	Remove food and water (15 h)*	SCT (1 h)*, Grouping (4 h), Strobe light (2 x 3 h) and light on (1 h)	Cage tilted 45° (14 h)	Wet bedding (14 h)
C	Cage tilted 45° (14 h)	Light on (3 h, 1h, 2 h), Strobe light (2 x 1h)	Wet bedding (14 h)	Remove food and water (15 h)*	SCT (1 h)*, Grouping (4 h), Strobe light (3 x 2 h) and light on (1 h)	Light on (3 h, 2 x 2 h)	Wet bedding (14 h)
A	Strobe light (6 h)	Wet bedding (14 h)	Light on (3 x 2 h)	Remove food and water (15 h)*	SCT (1 h)*, Grouping (4 h), Strobe light (2 x 2 h) and light on (1 h)	Cage tilted 45° (14 h)	Wet bedding (14 h)
D	Cage tilted 45° (14 h)	Light on (3 h), strobe light (5 h)	Wet bedding (14 h)	Remove food and water (15 h)*	SCT (1 h)*, Grouping (4 h), Strobe light (2 x 2 3) and light on (2 h)	Cage tilted 45° (14 h)	Wet bedding (14 h)
C	Cage tilted 45° (14 h)	Light on (3 h, 1h, 2 h), Strobe light (2 x 1h)	Wet bedding (14 h)	Remove food and water (15 h)*	SCT (1 h)*, Grouping (4 h), Strobe light (3 x 2 h) and light on (1 h)	Light on (3 h, 2 x 2 h)	Wet bedding (14 h)

* For CMS and non-stressed controls.

Table S3. Gradual food reduction regime prior to touchscreen pre-training. For every rat, baseline food intake was determined as an average of three *ad libitum* food consumption measurements. Parallel to food reduction, rats were habituated in their home cage to peanut butter (PB) as well as to bacon pellets (BP) used as reward in touchscreen (TS) testing.

Day of food reduction	1	2	3	4	5	6	7	8	TS 1
% of baseline food intake	95	95	90	90	85	80	80	75	75
Habituation to						BP	BP	PB	PB

Table S4. Characteristics of gene-specific real-time qPCR primers.

Gene Symbol	Gene Name	Accession No.[1]	Primer Sequence	Amplicon size[2]
**Reference genes**				
18s rRNA	18s subunit ribosomal RNA	M11188	(+) acggaccagagcgaaagcat<br>(-) tgtcaatcctgtccgtgtcc	310
ActB	Beta-actin	NM_031144	(+) tgtcaccaactgggacgata<br>(-) ggggtgttgaaggtctcaaa	165
CycA	Cyclophilin A	XM_345810	(+) agcactggggagaaaggatt<br>(-) agccactcagtcttggcagt	248
Gapdh	Glyceraldehyde-3-phosphate dehydrogenase	NM_017008	(+) tcaccaccatggagaaggc<br>(-) gctaagcagttggtggtgca	168
Hmbs	Hydroxy-methylbilane synthase	NM_013168	(+) tcctggctttaccattggag<br>(-) tgaattccaggtgagggaac	176
Hprt1	Hypoxanthine guanine phosphoribosyl transferase 1	NM_012583	(+) gcagactttgctttccttgg<br>(-) cgagaggtccttttcaccag	81
Rpl13A	Ribosomal protein L13A	NM_173340	(+) acaagaaaaagcggatggtg<br>(-) ttccggtaatggatctttgc	167
Ywhaz	Tyrosine 3-monooxygenase/tryptophan 5-monooxygenase activation protein, zeta	BC094305	(+) ttgagcagaagacggaaggt<br>(-) gaagcattggggatcaagaa	136
**Target genes**				
Bdnf	Brain-derived neurotrophic factor	NM_001270630.1	(+) gaaagtcccggtatcaaaag<br>(-) cgccagccaattctctttttg	183
Disc1	Disrupted in Schizophrenia 1	NM_175596.2	(+) agagagtgtggaaagcggac<br>(-) atgagattcctgcaaggggga	197
Spinophilin	protein phosphatase 1, regulatory subunit 9B (Ppp1r9b)	NM_053474	(+) tcctgtggagttggagaagg<br>(-) tgcctttggtgttcctaagc	239
Homer1	Homer scaffolding protein 1	NM_031707	(+) cacccgatgtgacacagaac<br>(-) tgttgcttccactgcttcac	220
Homer2	Homer scaffolding protein 2	NM_053309	(+) ctgccaggttagccagagac<br>(-) tcttcacattggcagcactc	219
Homer3	Homer scaffolding protein 3	NM_053310	(+) ggtcaaagaagctgccagac<br>(-) tgcggaacagcttctcttct	143
Shank 1		NM_031751	(+) cactctcagcacctggaaca<br>(-) gaagggtgtctgtccgttgt	177
Shank 2		NM_201350	(+) cctccaggactgcagagaac<br>(-) atttctgccttcgcatcgta	236
Shank 3		NM_021676	(+) ctgtgtggaggaagtgcaga<br>(-) gaacaaagccaaaaccctca	188
Cofilin 1		NM_017147	(+) gatgctgccagacaaggact<br>(-) cgggggcccagaaaatgaat	101
Nr3c1	Glucocorticoid receptor	NM_012576.2	(+) ggccggtcagtgttttctaa<br>(-) caatcgtttcttccagcaca	233
Nr3c2	Mineralocorticoid receptor	NM_013131.1	(+) tgagttccttcccacctgtc<br>(-) aagcctcatctccacacacc	192
Fkbp5	FK506 binding protein 5	XM_006256222.3	(+) gcactgaggaagcagaggttt<br>(-) gtctcctcactagtccccact	175
Nrg1	Neuroregulin 1	NM_001271118	(+) agcgaaaggtatgtatcagcca<br>(-) ggacacgggtggagacattt	111
Gsk3b	Glycogen synthase kinase 3 beta	NM_032080.1	(+) ccactcaagaactgtcaagtaacc<br>(-) tccacggtctccagcattag	131

[1] Genbank accession number of cDNA and corresponding gene, available at http://www.ncbi.nlm.nih.gov/

[2] Amplicon length in base pairs

Table S5. Target gene expression levels. Target genes were normalised to reference genes and calculated as % of the control group mean (for prefrontal cortex) or the control group mean of the dorsal hippocampus (for ventral and dorsal gene expression). For each gene, group mean (± SD) and sample size *n* (in brackets) are displayed. T – Main effect of treatment, H – Main effect of hedonic state, I –Interaction effect of hedonic state x treatment.

Target gene	Control	Anhedonic	Responder	Low-responder	Two-way ANOVA
			Prefrontal cortex		
Nr3c2	100.0 ± 16.7 (7)	90.6 ± 11.2 (9)	95.3 ± 10.5 (9)	90.9 ± 7.7 (7)	
Nr3c1	100.0 ± 16.9 (7)	99.9 ± 8.3 (9)	89.3 ± 9.6 (9)	94.4 ± 9.9 (7)	T:$F(1,27)=4.07$ $p=0.054$
Fkbp5	100.0 ± 73.0 (8)	140.8 ± 69.8 (10)	104.4 ± 55.9 (10)	108.1 ± 62.0 (8)	
Disc1	100.0 ± 49.3 (8)	111.9 ± 39.2 (10)	94.5 ± 67.5 (10)	108.7 ± 47.3 (8)	
Gsk3b	100.0 ± 10.4 (7)	108.8 ± 26.2 (9)	108.5 ± 13.8 (9)	98.6 ± 9.5 (7)	
Bdnf	100.0 ± 13.3 (7)	106.9 ± 18.4 (10)	96.7 ± 23.1 (9)	101.7 ± 8.4 (7)	
Nrg1	100.0 ± 9.7 (7)	113.6 ± 16.3 (10)	102.2 ± 11.8 (9)	102.9 ± 6.0 (7)	
Homer1	100.0 ± 43.5 (8)	121.1 ± 30.9 (10)	96.8 ± 36.5 (10)	104.0 ± 27.7 (8)	
Homer2	100.0 ± 14.9 (7)	103.6 ± 26.7 (10)	91.1 ± 9.5 (9)	96.9 ± 12.1 (7)	
Homer3	100.0 ± 16.5 (7)	111.1 ± 28.1 (9)	101.0 ± 10.7 (9)	96.0 ± 37.1 (8)	
Shank 1	100.0 ± 16.0 (7)	104.4 ± 10.4 (9)	95.1 ± 7.3 (9)	98.4 ± 13.2 (7)	
Shank 2	100.0 ± 15.1 (7)	93.4 ± 11.9 (9)	93.3 ± 9.0 (9)	87.6 ± 30.0 (8)	
Shank 3	100.0 ± 16.6 (7)	92.3 ± 8.3 (9)	95.7 ± 15.3 (9)	81.4 ± 26.9 (8)	
Spinophilin	100.0 ± 46.2 (8)	109.6 ± 25.5 (9)	99.3 ± 43.7 (10)	100.4 ± 42.1 (8)	
Cofilin 1	100.0 ± 45.2 (8)	137.1 ± 10.3 (10)	131.4 ± 9.1 (8)	130.1 ± 14.1 (7)	I:$F(1,28)=5.51$ $p=0.026$
			Dorsal Hippocampus		
Nr3c2	100.0 ± 19.4 (9)	79.2 ± 34.6 (10)	97.9 ± 44.7 (10)	77.6 ± 26.9 (8)	H:$F(1,33)=3.47$ $p=0.072$
Nr3c1	100.0 ± 39.9 (9)	90.0 ± 40.8 (10)	126.7 ± 42.5 (9)	98.3 ± 23.7 (7)	
Fkbp5	100.0 ± 34.6 (8)	97.1 ± 34.8 (10)	112.5 ± 34.4 (9)	91.2 ± 32.8 (8)	
Disc1	100.0 ± 29.4 (9)	93.7 ± 35.1 (10)	93.2 ± 50.1 (9)	96.7 ± 39.7 (8)	
Gsk3b	100.0 ± 6.3 (8)	100.9 ± 10.5 (10)	119.4 ± 34.5 (10)	92.0 ± 17.3 (8)	I:$F(1,32)=4.03$ $p=0.053$
Bdnf	100.0 ± 11.7 (8)	119.8 ± 12.9 (9)	124.7 ± 32.1 (9)	126.5 ± 25.0 (8)	T:$F(1,30)=3.87$ $p=0.058$
Nrg1	100.0 ± 36.5 (9)	79.0 ± 12.6 (8)	90.2 ± 18.1 (10)	98.1 ± 21.1 (8)	
Homer1	100.0 ± 38.3 (9)	90.6 ± 10.9 (9)	110.8 ± 25.9 (10)	97.2 ± 21.9 (8)	
Homer2	100.0 ± 24.7 (9)	82.6 11.8 (10)	103.3 ± 38.3 (10)	79.8 ± 21.0 (8)	H:$F(1,33)=5.63$ $p=0.024$
Homer3	100.0 ± 29.6 (9)	108.0 ± 24.6 (10)	101.0 ± 24.3 (10)	124.6 ± 34.1 (8)	
Shank 1	100.0 ± 47.2 (9)	108.9 ± 19.0 (9)	100.3 ± 42.3 (10)	94.9 ± 34.0 (8)	
Shank 2	100.0 ± 31.6 (9)	95.0 ± 25.1 (10)	92.2 ± 41.4 (10)	83.7 ± 25.2 (7)	
Shank 3	100.0 ± 14.7 (9)	86.6 ± 23.2 (10)	104.4 ± 19.0 (9)	95.5 ± 14.4 (7)	
Spinophilin	100.0 ± 34.0 (9)	117.9 ± 21.8 (9)	105.9 ± 22.7 (9)	109.7 ± 37.1 (8)	
Cofilin 1	100.0 ± 7.8 (8)	86.9 ± 25.3 (10)	88.5 ± 31.0 (10)	100.7 ± 6.5 (7)	
			Ventral Hippocampus		
Nr3c2	86.7 ± 16.3 (9)	81.0 ± 27.7 (10)	93.3 ± 26.2 (9)	73.1 ± 19.7 (8)	
Nr3c1	108.9 ± 13.8 (8)	101.0 ± 26.6 (9)	101.4 ± 28.9 (10)	93.5 ± 47.0 (8)	
Fkbp5	106.6 ± 19.1 (6)	108.5 ± 54.1 (9)	105.1 ± 43.6 (8)	112.2 ± 46.1 (8)	
Disc1	236.0 ± 163.7 (9)	167.5 ± 92.3 (9)	153.1 ± 78.2 (9)	261.8 ± 200.8 (8)	I:$F(1,31)=3.46$ $p=0.072$
Gsk3b	134.0 ± 39.0 (9)	119.1 ± 26.8 (10)	132.3 ± 38.6 (10)	105.7 ± 26.5 (8)	H:$F(1,33)=3.48$ $p=0.071$
Bdnf	102.0 ± 55.5 (9)	121.1 ± 36.0 (10)	109.1 ± 33.9 (10)	124.5 ± 28.7 (8)	
Nrg1	196.4 ± 110.0 (9)	162.1 ± 74.0 (9)	164.2 ± 73.7 (9)	187.6 ± 88.0 (8)	
Homer1	85.3 ± 14.3 (9)	86.0 ± 18.4 (10)	99.9 ± 12.7 (9)	74.2 ± 21.0 (8)	
Homer2	91.6 ± 16.4 (9)	89.3 ± 23.6 (10)	91.7 ± 32.8 (10)	89.0 ± 29.0 (8)	
Homer3	119.4 ± 50.7 (9)	123.0 ± 37.0 (10)	86.7 ± 25.4 (10)	108.8 ± 33.3 (8)	T:$F(1,33)=4.03$ $p=0.053$
Shank 1	155.9 ± 80.5 (9)	161.8 ± 90.9 (10)	145.0 ± 68.4 (9)	130.4 ± 65.7 (8)	
Shank 2	130.2 ± 49.8 (9)	115.9 ± 40.1 (9)	116.3 ± 20.7 (8)	132.9 ± 55.3 (8)	
Shank 3	133.6 ± 72.7 (9)	123.5 ± 66.4 (10)	93.2 ± 70.2 (10)	101.0 ± 37.8 (8)	
Spinophilin	129.4 ± 42.8 (9)	121.5 ± 38.7 (10)	110.8 ± 32.3 (10)	112.7 ± 26.6 (8)	
Cofilin 1	105.2 ± 16.7 (8)	95.5 ± 27.9 (10)	92.7 ± 34.6 (9)	105.8 ± 18.3 (8)	

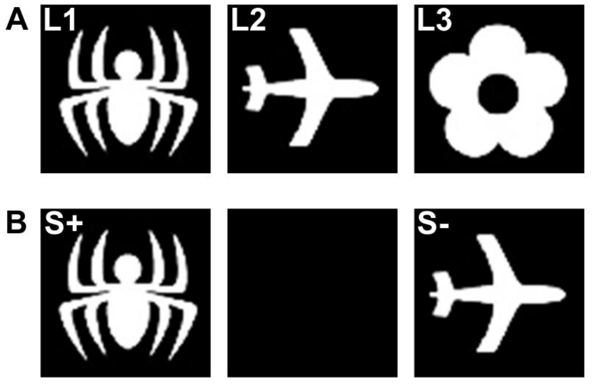

Figure S1. Object-location association pairs in the different paired-associates learning tasks. (A) The three symbols are shown in their correct location (L) on the touchscreen (Spider-L1, Plane-L2, Flower-L3). **(B)** An example trial of the six possible trial types. The spider is displayed in its correct location L1 (S+), whereas the plane is presented in an incorrect location (S-). The third window is blank.

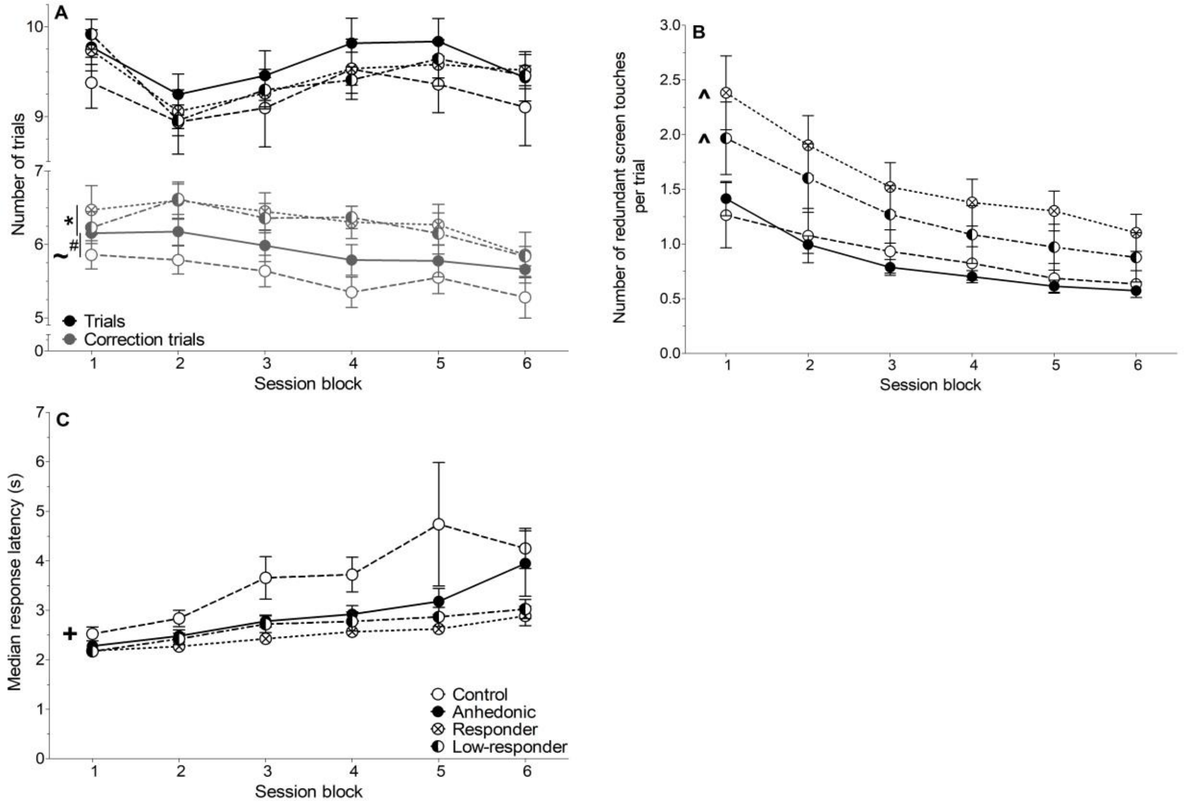

Figure S2. Touchscreen parameters within the course of an average session. (A) Number of trials (black) and correction trials (grey). **(B)** Number of redundant touches per trial (trial or correction trial). **(C)** Median response latency. Group means (± SEM) are displayed. Bonferroni post-hoc tests are indicated with **p* < 0.05, #*p* < 0.06, '+' indicating a significant difference of the respective group to the three other groups, '~' a significant difference to responder and low-responder or '^' to control and anhedonic-like rats (Bonferroni post-hoc comparisons). *N* = 10 per group.

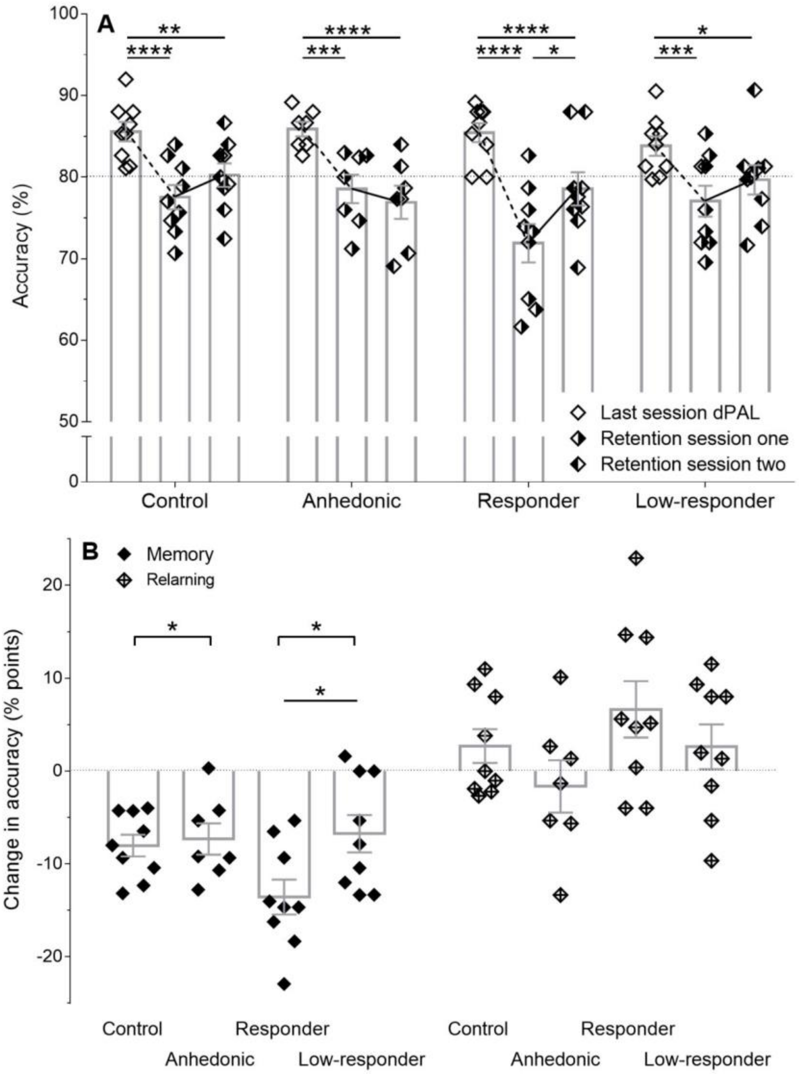

Figure S3. Retention of the dPAL task. (A) Accuracy for each rat including the last dPAL session during task acquisition and the first and second retention session after 10 days hiatus. The dotted line (···) indicates the drop in group accuracy due to the hiatus (memory performance) and the continuous line (—) presents the group relearning performance. **(B)** Individual memory and relearning performance are shown. Group means (± SEM) are displayed, main effect of hedonic state and post-hoc statistics are indicated by $****p < 0.0001$, $***p < 0.001$, $**p < 0.01$, $*p < 0.05$, $^{\#}p < 0.06$.

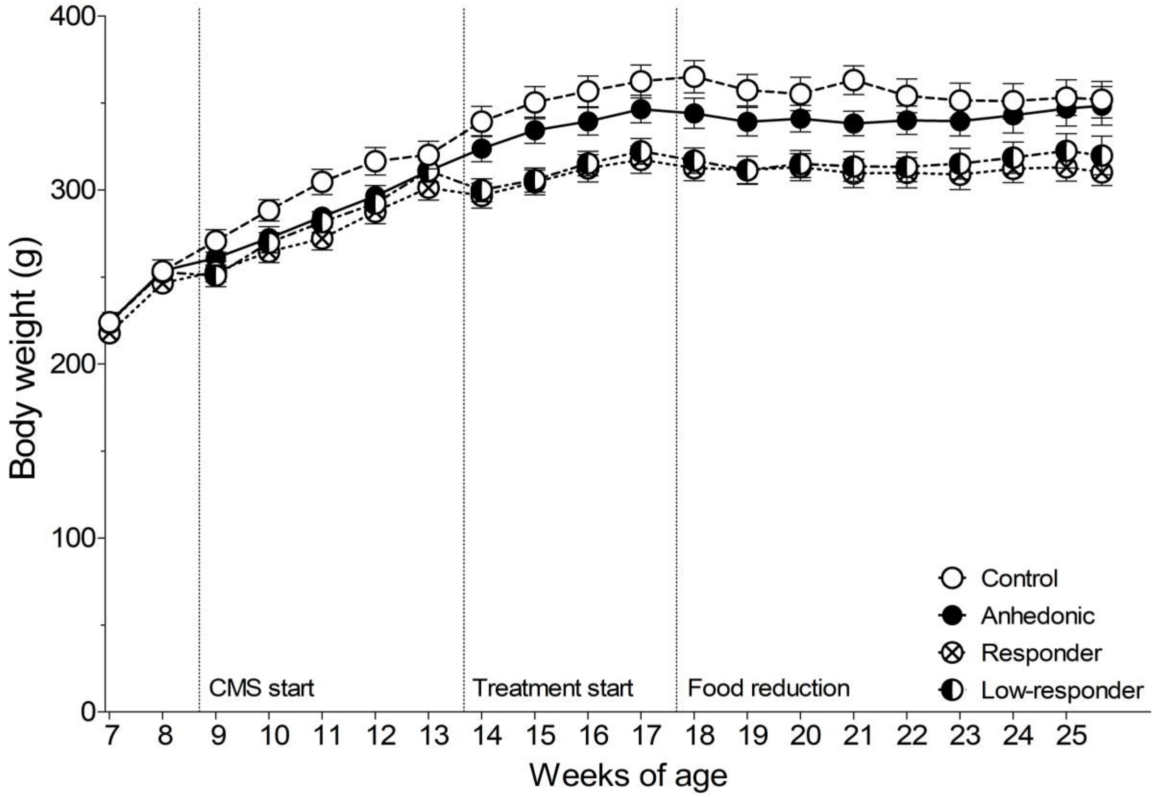

Figure S4. Body weight. Vertical lines indicate the beginning of the chronic mild stress (CMS) paradigm, followed by treatment with vortioxetine (responder & low-responder) or vehicle (control & anhedonic). Gradual food reduction preceded touchscreen pre-training and dPAL (different paired-associates learning) testing by 7 days. Group means (± SEM) are displayed; *n* = 10 per group.

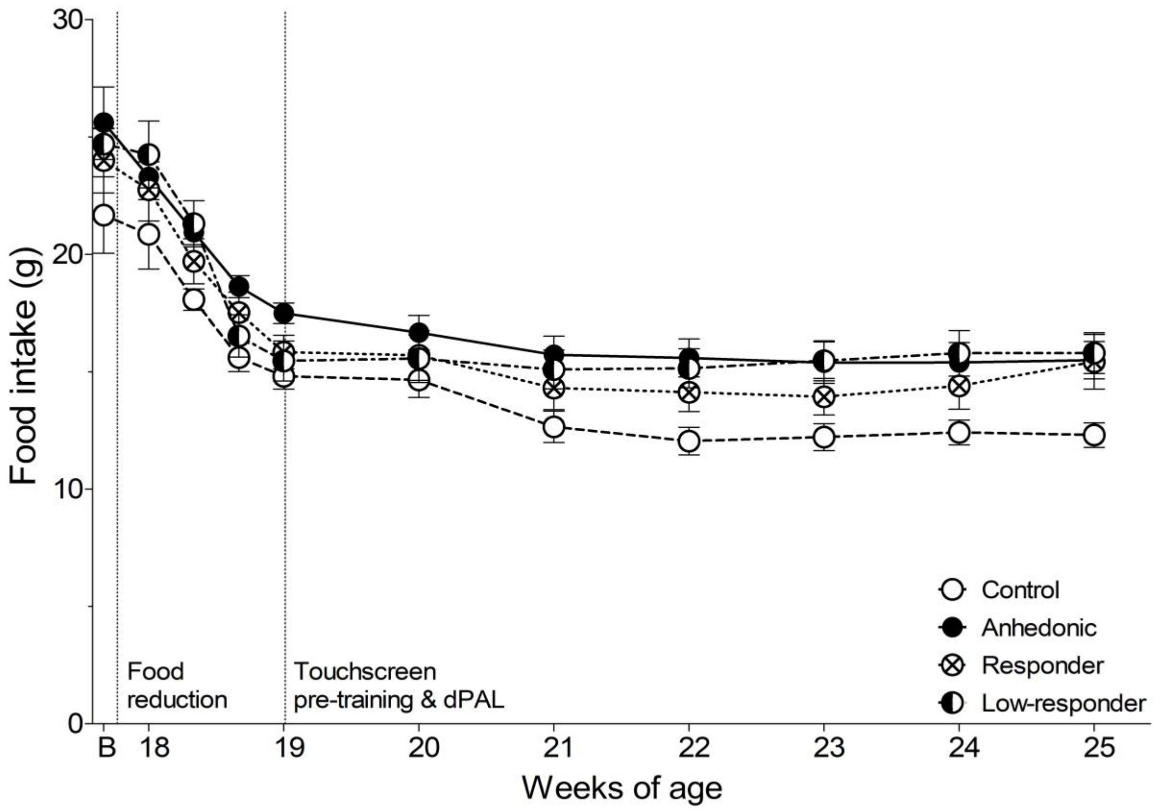

Figure S5. Food intake. Three *ad libitum* measurements averaged to one food intake baseline B. Gradual food reduction commenced before touchscreen pre-training and dPAL (different paired-associates learning) testing. Group means (± SEM) are displayed; *n* = 10 per group.
